# Drug interactions with QT-prolonging antibiotics: an epidemiological study in community pharmacies

**DOI:** 10.1186/2049-3258-73-S1-P42

**Published:** 2015-09-17

**Authors:** Eline Vandael, Chantal Leirs, Margaux Claes, Amelie Matheve, Elien Verbeeck, Delphine Demeyer, Veerle Foulon

**Affiliations:** 1 KU Leuven, Leuven, Belgium; 2 Escapo NV, Mechelen, Belgium; 3 2nd Master Pharmaceutical Care, Faculty of Pharmaceutical Sciences, KU Leuven, Leuven, Belgium; 4 Departement Pharmaceutical and Pharmacological Sciences, KU Leuven, Leuven, Belgium

## Background

More than 170 drugs of different therapeutic classes are linked with a prolongation of the QTc-interval, which in rare cases can lead to Torsade de Pointes (TdP) and sudden cardiac death. The risk is especially high in patients with other risks factors for QTc-prolongation or when two or more QTc-prolonging drugs are combined.

## Aims

To investigate the use of QT-prolonging antibiotics, concomitant risk factors for QT-prolongation and the current management of these interactions in community pharmacies.

## Methods

An epidemiological study on data of a dispensing database of Flemish community pharmacies (Surplus network; N=100) was performed. The patients who received a QT-prolonging antibiotic in the last week of May 2014 were selected and their medication histories (February-May 2014) were screened for other QT-prolonging drugs and concomitant risk factors (summarized in a risk score: = 65 years, female, cardiovascular disease, diabetes, thyroid disturbances, each 1 point; potassium-lowering diuretics: 3 points; antiarrhythmic drugs: 4 point). Furthermore, the management of QT-signals by the pharmacist was analyzed.

## Results

In the study period, 928 patients (56.4% females, median age 55.5 years) received a QT-prolonging antibiotic (especially azithromycin, cipro/moxifloxacin). Of these patients, 313 (33.7%) were synchronously treated with another QT-prolonging drug (of whom 67 patients with a drug with a known risk of TdP). Moreover, 107 of the 313 patients (34.2%) had a risk score =5. A drug-drug interaction signal only turned up in 5 (of the 67) patients (because the warning system was turned off in most pharmacies) and the general practitioner was only contacted in one of these cases.

## Conclusion

There is a high prevalence of interactions with QT-prolonging antibiotics in community pharmacies. However, the management and awareness of these interactions is limited. We are currently developing a decision support system to help pharmacists handling these interactions.

